# Which personality traits can mitigate the impact of the pandemic? Assessment of the relationship between personality traits and traumatic events in the COVID-19 pandemic as mediated by defense mechanisms

**DOI:** 10.1371/journal.pone.0251984

**Published:** 2021-05-19

**Authors:** Alessio Gori, Eleonora Topino, Letizia Palazzeschi, Annamaria Di Fabio

**Affiliations:** 1 Department of Health Sciences, University of Florence, Florence, Italy; 2 Department of Human Sciences, LUMSA University of Rome, Rome, Italy; 3 Department of Education, Languages, Interculture, Letters and Psychology (Psychology Section), University of Florence, Florence, Italy; Universidad Nacional de Educacion a Distancia, SPAIN

## Abstract

The COVID-19 pandemic has had a significant influence on the lives of people around the world and could be a risk factor for mental health diseases. This study aimed to explore the psychological impact of the COVID-19 pandemic by identifying patterns related to post-traumatic symptoms by considering personality and defensive styles. Specifically, it was hypothesized that neuroticism was negatively associated with impact of event, as opposed to extraversion, agreeableness, conscientiousness, and openness traits. The mediation role of mature, neurotic, and immature defenses in these relationships was also investigated. This study involved 557 Italian individuals (71.3% women, 28.7% men; *M*_*age*_ = 34.65, *SD* = 12.05), who completed an online survey including the Impact of Event Scale—Revised, Forty Item Defense Style Questionnaire (DSQ-40) and Ten Item Personality Inventory. Results showed a nonsignificant effect for extraversion and openness on impact of event. The negative influence of neuroticism was instead confirmed in a partial parallel mediation involving significant effects from immature and neurotic defenses in the indirect path. Finally, agreeableness and conscientiousness delineated two protective pathways regarding impact of event, determining two total parallel mediation models in which both these personality traits were negatively associated with immature defensive styles, and conscientiousness was also positively related to mature defenses. These findings provide an exploration post-traumatic symptom patterns during the COVID-19 pandemic, involving the big five personality traits and defense mechanisms. These results may be useful for developing interventions, treatments, and prevention activities.

## Introduction

The COVID-19 pandemic has had a profound influence on the lives of people around the world [1–3]. It has an impact not only on the sphere of physical health [4], but its effects extended to individual and collective levels in behavioral and economic areas [5, 6]. The protective measures adopted in countries to stem the spread of the pandemic, by requiring the adoption of new protective habits, have significantly impacted the world economy, causing many people to be in a state of financial instability and uncertainty about the future [7, 8]. This scenario had repercussions on organizations and the health of workers [9]. Indeed, all this could have a profound effect on mental health [10]. Both the restrictive measures adopted by governments and the spread of the virus itself were associated with lower levels of life satisfaction and wellbeing [11, 12] and with higher levels of anxiety [13–16], depression [17], anger [18], fear, and worry [18–20], resulting in fertile ground for the development of distress and chronic psychological symptoms in some people [21]. In fact, previous research has consistently identified symptoms of post-traumatic stress disorder (PTSD) [14, 22–25], which could last beyond the course of the pandemic. PTSD, in turn, was associated with poorer physical health, suicide attempts, and impairment in different areas of life [26, 27]. Regarding organizations, from a healthy business perspective [28], healthy organizations require healthy workers [29]. In this framework, this study aims to foster a better understanding of the effects of COVID-19 on mental health, identifying patterns related to post-traumatic symptoms by considering personality and defensive styles.

The psychological and behavioral responses to the pandemic can be influenced by several factors, including a person’s characteristics and resources [30, 31], as evidenced by previous research that has highlighted the significant influence of personality traits on reactions to stress [32, 33]. In this field, the Big Five Model of Costa and McCrae [34] is one of the most frequently used, in which five dimensions (extraversion, agreeableness, conscientiousness, neuroticism, and openness) represent a coherent and basically stable set of aspects that influence the affects, thoughts, and behaviors of individuals in their different life experiences. Among these, neuroticism appears to be a relevant risk factor in the development of post-traumatic stress disorder in problematic conditions [35]. Individuals with less emotional stability reported more intense and lasting emotional responses, associated with a tendency to perceive the impact of stressful events with greater intensity [36, 37]. Conversely, extroversion, openness, agreeableness, and conscientiousness have been associated with functional and active strategies for solving problems in difficult situations such as seeking support, positive reinterpretation, growth, and acceptance [38]. These dimensions have been consistently positively associated with subjective wellbeing and life satisfaction [39]. Personality traits, therefore, can shape an individual’s responses to life situations by influencing their cognitive assessments, the emotions associated with them, and the strategies used to regulate those affective activations [38, 40]. Other relevant factors in managing the psychological impact of stressful events are defenses, which can be defined as mechanisms that "mediate the individual’s reaction to emotional conflicts and external stressors" [41] (p. 844) and may be more or less adaptive depending on the context in which their occur. In other words, psychological health is not only linked to the application of mature defense strategies, but above all to the appropriate use of a variety of defenses based on the circumstances [42]. To confirm this, previous research has shown an inverse association between adaptive levels of defensive functioning and perceived distress, depressive, and post-traumatic symptoms during the COVID-19 pandemic [43, 44].

On this basis, the aim of this work is to contribute to the knowledge of the psychological impact of the COVID-19 pandemic. Therefore, a series of parallel mediation models were implemented to analyze the relationships between personality traits and post-traumatic symptoms. More specifically, it was hypothesized that neuroticism was negatively associated with impact of event, contrary to the traits of extraversion, agreeableness, conscientiousness, and openness, for which a positive relationship is assumed. The mediation role of mature, neurotic, and immature defenses in these pathways was also explored.

## Method

### Participants and procedures

The participants in this study were 557 Italian individuals (ages 18–88 years; *M* = 34.65, *SD* = 12.05), 397 of which were woman (71.3%) and 160 were man (28.7%). They were recruited during the COVID-19 pandemic on the internet by circulating a link to a survey administered through the Google Forms platform. The survey was launched on March 24th, 2020 and remained open until March 31st, 2020. Before starting, participants were informed about the general aim of the study and provided with informed consent electronically. Data was anonymously collected, and the privacy of the respondents was guaranteed. Participants did not receive compensation for being involved in the study and were free to withdraw at any moment. The research protocol was reviewed and approved by the Ethical Committee of the Integrated Psychodynamic Psychotherapy Institute (IPPI).

### Measures

#### Impact of Event Scale—Revised (IES-R)

The Impact of Event Scale—Revised (IES-R) is a self-report measure developed by Weiss and Marmar [45] to assess the level of post-traumatic symptomatology resulting from a traumatic event. It is composed of 22 items rated on a five-point Likert scale ranging from 0 (*not at all*) to 4 (*extremely*). In addition to a total score, it also allows for evaluation of three subdimensions: intrusion, avoidance, and hyperarousal. The Italian version, previously validated by Craparo and colleagues [46], was used in this study. It has satisfactory psychometric properties, showing adequate internal consistency for each subscale (intrusion, *α* = .78; avoidance, *α* = .72; hyperarousal, *α* = .83) [46], as well as excellent Cronbach’s α for the total score (*α* = .95) in Italian populations during the COVID-19 pandemic [47].

#### Forty Item Defense Style Questionnaire (DSQ-40)

The Forty Item Defense Style Questionnaire (DSQ-40) was developed by Andrews and colleagues [48] to assess the degree to which the respondent uses mature, neurotic, or immature defense mechanisms. It included 40 items on a nine-point scale from 1 (*strongly disagree*) to 9 (s*trongly agree*). In the present study, the Italian version, previously validated by Farma and Cortinovis [49] and showing acceptable psychometric properties (mature defense: *α* = .61; neurotic defense: *α* = .59; immature defense: *α* = .80), was administered.

#### Italian Ten Item Personality Inventory (I-TIPI)

The Ten Item Personality Inventory (TIPI) was developed by Gosling and colleagues [50] to assess personality traits in terms of the big five model [51]. It includes 10 items on an eight-point scale ranging from 0 (*disagree strongly*) to 7 (*agree strongly*). It allows for the evaluation of five personality traits: extraversion, agreeableness, conscientiousness, neuroticism, and openness. In this study, the Italian version by Di Fabio, Gori, and Giannini [52] was used, showing satisfactory psychometric properties (from *α* = .78 for agreeableness, to *α* = .82 for extraversion) [52].

### Data analysis

SPSS statistical software (v. 25.0 for windows) was used to analyze the collected data. First, means and standard deviations for all scales were calculated. Then, a Pearson’s correlation analysis was implemented to evaluate the associations between the variables under study. To assess the effect of the different personality traits on impact of event during the COVID-19 pandemic, while exploring the role of the defense mechanisms in this relationship, several parallel mediation models were tested using the macro-program PROCESS 3.4 [53]. The output variable of these models was the total score of the IES-R, although the scale also has sub-dimensions, as it includes partial scores and therefore guarantees a broader detection of the extent of the event. For each regression coefficient included in the models, the 95% confidence interval (CI) was calculated. Finally, the statistical relevance of the indirect effects was verified by performing the bootstrap technique for each of the 5,000 bootstrapped samples within 95% of the confidence interval.

## Results

Means and standard deviations of the measures and Pearson’s correlation matrix are shown in Table 1.

**Table 1:** Correlations, means and standard deviations of the variables.

	1	1.1	1.2	1.3	2.1	2.2	2.3	3.1	3.2	3.3	3.4	3.5	*M*	*SD*
1. Impact of event	1												33.05	16.75
1.1 Intrusion	.844**	1											11.52	6.06
1.2 Avoidance	.932**	.650**	1										12.18	7.18
1.3 Hyperarousal	.906**	.625**	.826**	1									9.35	5.46
2.1 Mature defenses	-.016	.059	-.037	-.066	1								42.89	9.47
2.2 Neurotic defenses	.394**	.347**	.376**	.330**	.278**	1							34.30	10.07
2.3 Immature defenses	.396**	.361**	.347**	.358**	.276**	.526**	1						95.18	25.91
3.1 Extraversion	.003	.026	-.011	-.005	.126**	-.002	-.029	1					7.65	3.27
3.2 Agreeableness	-.118**	**-.065**	-.094*	-.166**	.058	.051	-.302**	-.221**	1				9.95	2.42
3.3 Conscientiousness	-.090*	-.056	-.101*	-.081	.157**	-.083	-.211**	.011	.125**	**1**			10.56	2.58
3.4 Neuroticism	.457**	.327**	.424**	.481**	-.220**	.219**	.332**	-.060	-.273**	-.337**	1		7.84	3.12
3.5 Openness	-.049	-.063	-.041	-.028	.090*	-.022	-.112**	.258**	.010	-.027	-.020	1	12.18	7.18

Note:

**. Correlation is significant at the .01 level (2-tailed).

*. Correlation is significant at the .05 level (2-tailed).

Results highlighted significant and positive associations of impact of event with neurotic defenses (*r* = .394, *p* < .01), immature defenses (*r* = .396, *p* < .01), and neuroticism (*r* = .457, *p* < .01), as well as negative significant relationships with agreeableness (*r* = -.118, *p* < .01) and conscientiousness (*r* = -.090, *p* < .05). Furthermore, immature defenses were negatively and significantly correlated with agreeableness (*r* = -.302, *p* < .01), conscientiousness (*r* = -.211, *p* < .01), and openness (*r* = -.112, *p* < .01), while they were positively and significantly related to neuroticism (*r* = .332, *p* < .01). The mature defenses scale was significantly and positively correlated with conscientiousness (*r* = .157, *p* < .01) and openness (*r* = .090, *p* < .05) and showed a significant and negative association with neuroticism (*r* = -.220, *p* < .01). Finally, a significant and positive relation was found between neuroticism and neurotic defenses (*r* = .219, *p* < .01).

A series of parallel mediations was performed to investigate the contributions of the different to the effects between personality traits and impact of event (see Table 2). The results showed that the effect of extraversion on impact of event was nonsignificant (β = .00, *p* = .948; LLCI = -.412—ULCI = .441), as was for openness (β = -.050, *p* = .244; LLCI = -.874—ULCI = .223).

**Table 2:** Models effect indices.

Independent variable	Parallel mediators	Dependent variable	Total effect	Direct effect	Indirect effect
					[95% CI
					indirect effect]
Extraversion	Mature defenses	Impact of event	.003	.035	-.032
	Neurotic defenses				[-.392; .054]
	Immature defenses				
Agreeableness	Mature defenses	Impact of event	-.118**	-.041	-.077
	Neurotic defenses				[-.897; -.187]
	Immature defenses				
Conscientiousness	Mature defenses	Impact of event	-.090*	.026	-.116
	Neurotic defenses				[-1.046; -.471]
	Immature defenses				
Neuroticism	Mature defenses	Impact of event	.457***	.334***	.123
	Neurotic defenses				[.407; .939]
	Immature defenses				
Openness	Mature defenses	Impact of event	-.050	.006	-.056
	Neurotic defenses				[-.658; -.079]
	Immature defenses				

Note:

*p < 0.05.

**p < 0.01.

***p < 0.001.

**Fig 1:**
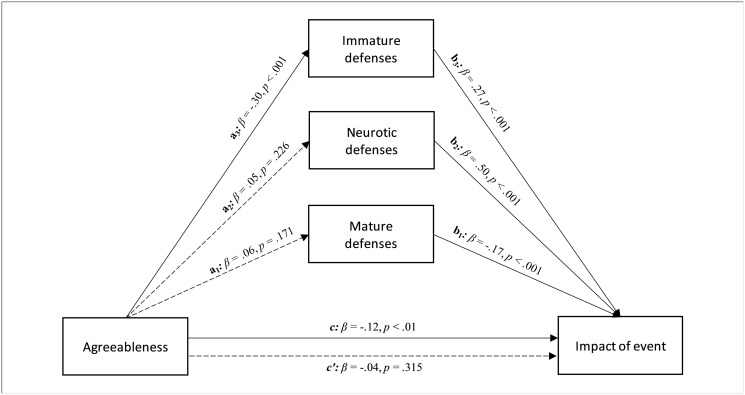
The relationship between agreeableness and impact of event, with different levels of defenses as parallel mediators: A parallel mediation model.

On the other hand, the data showed that agreeableness had a significant and negative effect on impact of event (path *c* in Fig 1; β = -.12, *p* < .01; LLCI = -1.392—ULCI = -.244). Furthermore, the agreeableness trait had an nonsignificant effect on both mature (path *a*_*1*_ in Fig 1; β = .06, *p* = .171; LLCI = -.099—ULCI = .554) and neurotic defenses (path *a*_*2*_ in Fig 1; β = .05, *p* = .226; LLCI = -.133—ULCI = .561), but it significantly and negatively affected immature defenses (path *a*_*3*_ in Fig 1; β = -.30, *p* < .001; LLCI = -4.086—ULCI = -2.382), which in turn were positively associated with impact of event (path *b*_*3*_ in Fig 1; β = .27, *p* < .001; LLCI = .115—ULCI = .238). Entering the three defensive styles in the model parallelly, only immature defenses played a statistically significant role in the relationship between agreeableness and impact of event, at a level whose direct effect was nonsignificant after controlling the mediators (path *c’* in Fig 1; β = -.04, *p* = .315; LLCI = -.839—ULCI = .271). Therefore, a total mediation occurred (*R*^*2*^ = .234, *F*(4, 552) = 42.158, *p* < .001; see Fig 1). The bootstrap procedure confirmed the statistical relevance of this indirect effect (Boot LLCI = .112- Boot ULCI = .240).

**Fig 2:**
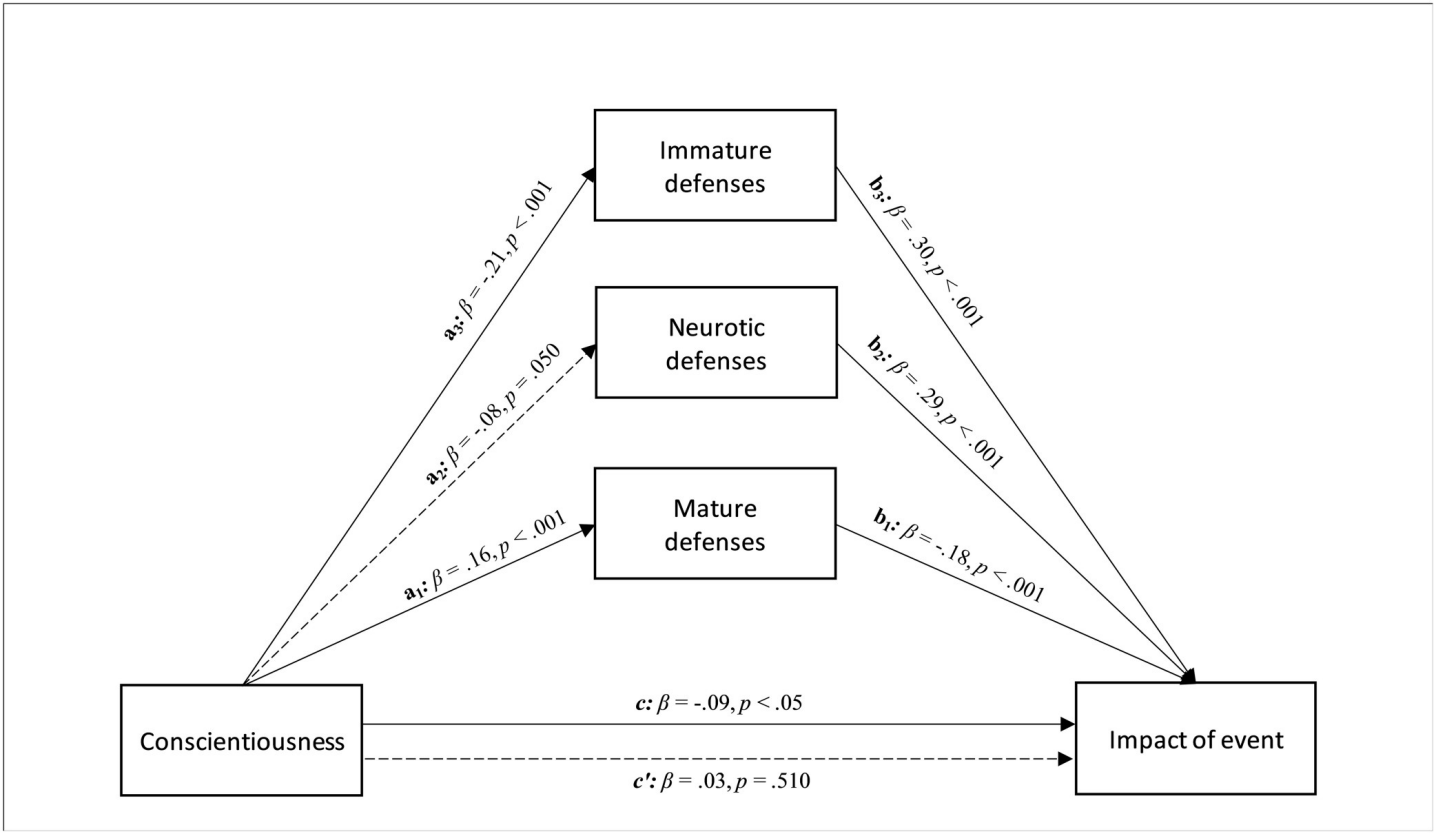
The relationship between conscientiousness and impact of event, with different levels of defenses as parallel mediators: A parallel mediation model.

The parallel mediation model involving conscientiousness highlighted its significant and negative total effect on impact of event (path *c* in Fig 2; β = -.09, *p* < .05; LLCI = -1.122—ULCI = -.045). Conscientiousness was also significantly and positively related to mature defenses (path *a*_*1*_ in Fig 2; β = .16, *p* < .001; LLCI = .272—ULCI = .876) and significantly and negatively associated with immature defenses (path *a*_*3*_ in Fig 2; β = -.21, *p* < .001; LLCI = -2.927—ULCI = -1.293), while showing a nonsignificant effect on neurotic defenses (path *a*_*2*_ in Fig 2; β = -.08, *p =* .05; LLCI = -.647—ULCI = -.000). On the other hand, the effect of mature defenses on impact of event was significant (path *b*_*1*_ in Fig 2; β = -.18, *p* < .001; LLCI = -.464—ULCI = -.184), as well as that of the immature defenses (path *b*_*3*_ in Fig 2; β = .30, *p* < .001; LLCI = .136—ULCI = .252). Entering the three defensive styles in the model parallelly, mature and immature defenses played a significant role in the relationship between conscientiousness and impact of the event, at a level whose direct effect became nonsignificant after controlling the mediators (path *c’* in Fig 2; β = .03, *p* = .511; LLCI = -.332—ULCI = .666). Therefore, a total mediation occurred (*R*^*2*^ = 0.233, *F*(4, 552) = 41.969, *p* < .001; see Fig 2). The bootstrap procedure confirmed the statistical relevance of this indirect effect (Boot LLCI = .132- Boot ULCI = .252).

**Fig 3:**
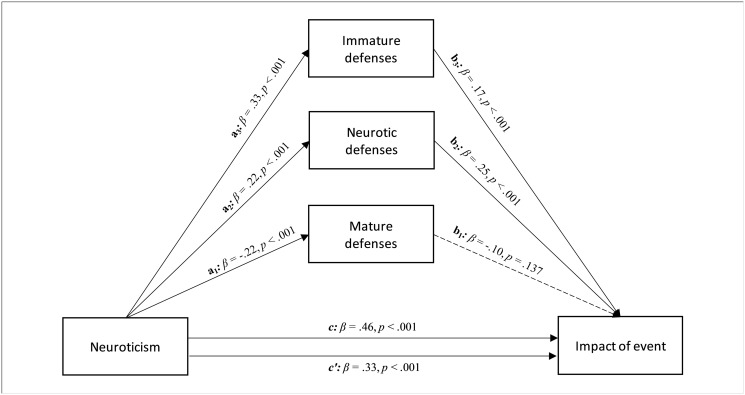
The relationship between neuroticism and impact of event, with different levels of defenses as parallel mediators: A parallel mediation model.

Finally, the personality trait of neuroticism showed a significant and positive total effect on impact of event (path *c* in Fig 3; β = .46, *p* < .001; LLCI = 2.058—ULCI = 2.855). Furthermore, neuroticism had significant effects on mature (path *a*_*1*_ in Fig 3; β = -.22, *p* < .001; LLCI = -.915—ULCI = -.420), neurotic (path *a*_*2*_ in Fig 3; β = .22, *p* < .001; LLCI = .445—ULCI = .971), and immature defenses (path *a*_*3*_ in Fig 3; β = .33, *p* < .001; LLCI = 2.102—ULCI = 3.410). In turn, the neurotic style was significantly related to impact of event (path *b*_*2*_ in Fig 3; β = .25, *p* < .001; LLCI = .273—ULCI = .559), as well as the immature defenses (path *b*_*3*_ in Fig 3; β = .17, *p* < .001; LLCI = .055—ULCI = .168). Entering the three defensive styles in the model parallelly, mature and immature defenses played a significant role in the relationship between conscientiousness and impact of event, albeit remaining significant after controlling the mediators (path *c’* in Fig 3; β = .33, *p* < .001; LLCI = 1.367—ULCI = 2.211). Therefore, a total mediation occurred (*R*^*2*^ = 0.318 *F*(4, 552) = 64.447, *p* < .001; see Fig 3). The bootstrap procedure confirmed the statistical relevance of this indirect effect (Boot LLCI = .053- Boot ULCI = .168).

The coefficients of the significant parallel mediation models are summarized in Table 3.

**Table 3:** Coefficients of the mediation models.

**Parallel mediation of different levels of defenses on the relationship between Agreeableness and Impact of event**																
	Consequent															
		M1				M2				M3				Y		
Antecedent		Coeff.	SE	*p*		Coeff.	SE	*p*		Coeff.	SE	*p*		Coeff.	SE	*p*
X1	*a*^*1*^	.228	.166	.171	*a*^*2*^	.214	.177	.226	*a*^*3*^	-3.234	.434	< .001	*c’*	-.284	.282	.315
M1		-	-	-		-	-	-		-	-	-	*b*^*1*^	-.305	.070	< .001
M2		-	-	-		-	-	-		-	-	-	*b*^*2*^	.470	. 076	< .001
M3		-	-	-		-	-	-		-	-	-	*b*^*3*^	.177	. 031	< .001
Constant	*i*_*M1*_	40.626	1.701	< .001	*i*_*M2*_	32.163	1.809	< .001	*i*_*M2*_	127.371	4.443	< .001	*i*_*Y*_	15.010	4.529	.001
		*R*^*2*^ = 0.003				*R*^*2*^ = 0.003				*R*^*2*^ = 0.091				*R*^*2*^ = 0.234		
		*F*(1, 555) = 1.876, *p =* .171				*F*(1, 555) = 1.472, *p =* .226				*F*(1, 555) = 55.581, *p <* .001				*F*(4, 552) = 42.158, *p <* .001		
**Parallel mediation of different levels of defenses on the relationship between Conscientiousness and Impact of event**																
	Consequent															
		M1				M2				M3				Y		
Antecedent		Coeff.	SE	*p*		Coeff.	SE	*p*		Coeff.	SE	*p*		Coeff.	SE	*p*
X2	*a*^*1*^	-.574	.154	< .001	*a*^*2*^	-.323	.165	.050	*a*^*3*^	-2.110	.416	< .001	*c’*	.167	.254	.022
M1		-	-	-		-	-	-		-	-	-	*b*^*1*^	-.324	.071	< .001
M2		-	-	-		-	-	-		-	-	-	*b*^*2*^	.481	. 074	< .001
M3		-	-	-		-	-	-		-	-	-	*b*^*3*^	.194	. 030	< .001
Constant	*i*_*M1*_	36.833	1.670	< .001	*i*_*M2*_	37.710	1.791	< .001	*i*_*M2*_	117.457	4.521	< .001	*i*_*Y*_	10.220	4.294	.018
		*R*^*2*^ = 0.025				*R*^*2*^ = 0.007				*R*^*2*^ = 0.044				*R*^*2*^ = 0.233		
		*F*(1, 555) = 13.946, *p <* .001				*F*(1, 555) = 3.853, *p =* .050				*F*(1, 555) = 25.735, *p <* .001				*F*(4, 552) = 41.969, *p <* .001		
**Parallel mediation of different levels of defenses on the relationship between Neuroticism and Impact of event**																
	Consequent															
		M1				M2				M3				Y		
Antecedent		Coeff.	SE	*p*		Coeff.	SE	*p*		Coeff.	SE	*p*		Coeff.	SE	*p*
X3	*a*^*1*^	-.667	.126	< .001	*a*^*2*^	.708	.134	< .001	*a*^*3*^	2.756	.333	< .001	*c’*	1.789	.215	< .001
M1		-	-	-		-	-	-		-	-	-	*b*^*1*^	-.104	.070	.137
M2		-	-	-		-	-	-		-	-	-	*b*^*2*^	.411	. 070	< .001
M3		-	-	-		-	-	-		-	-	-	*b*^*3*^	.111	. 029	< .001
Constant	*i*_*M1*_	48.121	1.062	< .001	*i*_*M2*_	28.746	1.128	< .001	*i*_*M2*_	73.582	2.807	< .001	*i*_*Y*_	-1.172	3.551	.742
		*R*^*2*^ = 0.048				*R*^*2*^ = 0.048				*R*^*2*^ = 0.332				*R*^*2*^ = 0.318		
		*F*(1, 555) = 28.110, *p <* .001				*F*(1, 555) = 28.019, *p <* .001				*F*(1, 555) = 68.548, *p <* .001				*F*(4, 552) = 64.447, *p <* .001		

***Note***: X1 = Agreeableness; X2 = Conscientiousness; X3 = Neuroticism; M1 = Mature defenses; M2 = Neurotic defenses; M3 = Immature defenses; Y = Impact of event

## Discussion

The COVID-19 pandemic is a global emergency that represents a significant risk not only for physical health [4], economic conditions, and healthy organizations [7, 29] but also for the psychological health of individuals. This is due to its numerous direct and indirect consequences in the psychological and social spheres [8] that could persist even after the pandemic ends [54]. Therefore, this study aimed to explore the pathways leading to post-traumatic symptoms by investigating the big five personality traits and their interactions with mature, neurotic, and immature defenses in their association with impact of event.

As expected, the results highlighted that neuroticism was the personality trait with the strongest total effect on impact of event, showing a significant positive association both directly and indirectly. Indeed, previous studies during the pandemic have found significant associations between this personality trait and generalized anxiety, depressive symptoms, worries, and pessimism related to COVID-19 [15, 55]. In line with this, other research has shown that people with high levels of neuroticism tend to react with intense negative emotional responses to frustration or loss, report worsening mental health conditions after stressful events, and were more at risk of post-traumatic symptoms [36, 37, 56, 57]. Furthermore, the data have shown that neuroticism also had an indirect effect on the impact of event through the significant influence of immature and neurotic defense mechanisms. This appears to be consistent with previous studies [58] that showed lower psychological adaptation skills in subjects with this personality trait during the pandemic. This was expressed through negative responses to stress linked to a lower level of resilience, which in turn, in other traumatogenic contexts, has shown a negative association with post-traumatic symptoms [59, 60].

The data also showed two protective pathways that involved agreeableness and conscientiousness, which interacted directly with defense mechanisms and only indirectly with impact of event. Specifically, both these personality traits were negatively associated with immature defensive styles, which represented a risk factor for mental health [61, 62]; furthermore, conscientiousness was positively related to mature defenses. Both of these indirect paths limited the level of impact of event, in line with the evidence in existing scientific literature about the positive association of agreeableness and conscientiousness with subjective wellbeing [39]. Indeed, previous research showed a negative relationship of agreeableness with anxiety and depression during the pandemic [63], while individuals with conscientiousness traits demonstrated greater compliance with prevention guidelines related to the COVID-19 [64]. Taken together, these results suggest the positive influence of these dispositions on the tendency to use effective strategies to face difficulties [65].

Finally, contrary to expectations, neither extraversion nor openness showed a significant association with impact of event. These results could be an expression of the specific situation involving COVID-19. Indeed, although previous research showed an association of extraversion and openness with higher levels of positive emotions and subjective wellbeing [39], the restrictions and preventive measures implemented to stem the pandemic could have attenuated the use of functional strategies usually associated with these personality traits, without going so far as to make them maladaptive. In other words, the level of adaptation can vary depending on the environment and the historical period [66].

These results need to be interpreted with caution, because of the limitations that should be considered. First, the participants in this study may not have been representative of the general population (e.g., they were recruited online, and this excluded people who did not have internet access). Secondly, a sectional design was used to implement this research, which does not allow for causal inference. Furthermore, the socioeconomic status of the participants was not explored, and this could be an interesting future challenge in light of previous evidence concerning the association between economic stratification and distress due to COVID-19 [e.g., 67]. In association with that, detailed information on the "remote" or "in presence" working condition and about the type of job was not collected: this can significantly affect the development of PTSD (especially with regards to health professionals who during the lockdown continued to work intensely while in contact with patients) [68] or, for example, enhance different perception of contagion risk between individuals with consciousness traits or, on the opposite, with higher levels of neuroticism.

Future research could overcome these limitations by implementing a longitudinal design, using a paper-pencil administration technique, and with a more comprehensive sample in which the differences in the psychological effect of COVID-19 may be explored, while considering socioeconomic and working conditions.

## Conclusion

The results of this research highlighted the association between the big five personality traits, defense mechanisms, and impact of event during the COVID-19 pandemic. More specifically, agreeableness and conscientiousness proved to be factors that may favor a more functional use of defense mechanisms. They were also negatively associated with the presence of post-traumatic symptoms, the opposite of what emerged for the neurotic trait. The understanding of the pathways involved in psychological distress during the pandemic may have practical implications for providing effective assistance to the population in terms of both individuals in personal contexts and workers in organizational contexts. This study can contribute by proposing differentiated interventions and treatments, starting from a better understanding of the defensive strategies used in relation to dispositional aspects. In this regard, these data suggest the usefulness of intervening to increase and support the use of mature defensive styles for dealing with the stressful experiences related to COVID-19 [69]. Treatments could focus, for example, on favoring increases in mentalizing or insight levels, which were positively associated with functional defenses and inversely with maladaptive defensive mechanisms [70, 71], as well as related to higher levels of mental health, meaningfulness, and satisfaction [72–74]. Finally, the psychological vulnerability of subjects with the trait of neuroticism was highlighted [15, 55], underlining the need to place a greater focus on both intervention and preventive perspectives in different life contexts. In this regard, it may be functional to intervene to limit the emotional dysregulation characterizing this trait, for example by implementing treatments focused on reducing alexithymia, which is negatively associated with the use of mature defenses [75], and was related to poorer mental health in both clinical and non-clinical subjects [76–78].
